# The Anti-Inflammatory Effect of *Humulus lupulus* Extract In Vivo Depends on the Galenic System of the Topical Formulation

**DOI:** 10.3390/ph15030350

**Published:** 2022-03-14

**Authors:** Zita Hurth, Marie-Luise Faber, Fabian Gendrisch, Martin Holzer, Birgit Haarhaus, Anja Cawelius, Kay Schwabe, Christoph Mathis Schempp, Ute Wölfle

**Affiliations:** 1 Research Center Skinitial, Department of Dermatology, Medical Center, University of Freiburg, Faculty of Medicine, University of Freiburg, 79106 Freiburg, Germany; zitahurth@aim.com (Z.H.); himarie@web.de (M.-L.F.); fabian.gendrisch@uniklinik-freiburg.de (F.G.); birgit.haarhaus@uniklinik-freiburg.de (B.H.); christoph.schempp@uniklinik-freiburg.de (C.M.S.); 2 Department of Pharmaceutical Technology and Biopharmacy, Institute of Pharmaceutical Sciences, Faculty of Chemistry and Pharmacy, University of Freiburg, 79104 Freiburg, Germany; martin.holzer@pharmazie.uni-freiburg.de; 3 Flavex Naturextrakte GmbH, 66780 Rehlingen, Germany; ac@flavex.com; 4 BSI-Beauty Science Intelligence GmbH, 30855 Langenhagen, Germany; k.schwabe@bsi-cosmetics.de

**Keywords:** *Humulus lupulus*, Franz diffusion cells, UVB erythema test

## Abstract

We demonstrated the anti-inflammatory and anti-oxidative effects of *Humulus lupulus* (HL) extract on solar simulator-irradiated primary human keratinocytes (PHKs) by analyzing ERK and p38 MAPK phosphorylation and production of IL-6 and IL-8. The anti-inflammatory effect of topically applied HL was further tested in vivo on human skin. To this end, we developed an oil-in-water (O/W) and a water-in-oil (W/O) cream with a lipid content of 40%. The anti-inflammatory effect of 1% HL extract incorporated in these two vehicles was assessed in a randomized, prospective, placebo controlled, double-blind UVB erythema study with 40 healthy volunteers. Hydrocortisone acetate (HCA) in the corresponding vehicle served as positive control. Surprisingly, both HL and HCA were only effective in the O/W system but not in the W/O formulation. Release studies using vertical diffusion cells (Franz cells) revealed that HCA was released in much higher amounts from the O/W cream compared to the W/O formulation. In summary, we have shown that 1% HL extract exerts anti-inflammatory effects comparable to 1% HCA, but only when incorporated in our O/W cream. Our findings confirm the critical role of the vehicle in topical anti-inflammatory systems.

## 1. Introduction

Our previous in vitro and ex vivo experiments revealed that *Humulus lupulus* (HL, hop) extract is a promising anti-psoriatic agent with prominent anti-proliferative effects [1]. Because epidermal barrier defects and scaling are pathogenetic key factors in psoriasis, the current guidelines and therapeutic concepts for psoriasis recommend a topical therapy with lipid-rich creams [2]. Similarly, other chronic inflammatory skin conditions with reduced lipid and ceramide content, such as atopic eczema, also benefit from a lipid-rich cream [3,4,5]. While lipophilic vehicles are advantageous for dry skin conditions, hydrophilic vehicles are more suitable for acute, oozing skin conditions. The most common vehicles with a very high lipid content are water-in-oil (W/O) creams with a hydrophilic inner phase dispersed in a lipophilic outer phase [6]. However, topical formulations with a similar high lipid content may also be obtained with lipid-rich oil-in-water (O/W) creams [7].

Hop has been cultivated by man since the early Middle Ages. Today, it is used primarily as a bitter flavoring and stabilizing agent in beer and as a mild, well-tolerated seda-tive and sleep-inducing agent, often in combination with other sedative medicinal plants, such as valerian [8]. The most important active ingredients are contained in the so-called lupulin glands in the female hop cones. The active compounds include lipophilic components such as essential oils and bitter acids. Myrcene is the major constituent of the hop essential oil with a share of up to 70% by volume [9]. It protects UVB-treated human dermal fibroblasts from photoaging by reducing the expression of reactive oxygen species (ROS), matrix metalloproteinase (MMP)-1, MMP-3, interleukin (IL)-6 and by increasing transforming growth factor (TGF)-β and Type I procollagen secretion. Therefore, myrcene is used in sunscreens or in anti-aging products [10,11]. Hop bitter acids make up 30% of the secondary metabolites in the lupulin glands [8,12,13]. The bitter acids consist of α-acids (humulone and its derivatives) and β-acids (lupulone and its derivatives). They act as radical scavengers and inhibit lipid peroxidation [14]. Humulone and lupulone display antibiotic effects against many Gram-positive bacteria (*Bacillus, Lactobacillus, Micrococcus, Streptococcus,* and *Staphylococcus*), whereas Gram-negative bacteria are either resistant or are only inhibited by very high concentrations [15,16]. Dermatologically important bacteria, such as *Propionibacterium acnes* or *Staphylococcus aureus*, which are involved in the formation of pustules in acne, are also inhibited by hop bitter acids [17].

A formulation containing humulone had an anti-tumor effect when applied topically to skin tumors chemically induced by TPA (12-O-tetradecanoylphorbol-13-acetate) in mice. This effect was mediated by suppressing the activation of nuclear factor ‘kappa-light-chain-enhancer’ of activated B-cells (NF-κB) and inhibiting cyclooxgenase-2. Furthermore, humulone inhibited the activation of the MAPK-pathway and the catalytic activity of ERK1/2 [18]. Humulone and lupulone also act as anti-angiogenic agents as demonstrated in vivo in a chicken chorioallantoic membrane model or in human umbilical vein endothelial cells [19,20].

In the present paper, we firstly assessed the anti-inflammatory effect of a humulone- and lupulone-enriched supercritical CO_2_ extract of *Humulus lupulus* (HL) in vitro using solar simulator-irradiated primary human keratinocytes (PHKs). Secondly, we conducted a randomized, prospective, placebo-controlled double-blind UVB erythema study with 40 healthy volunteers to investigate the anti-inflammatory effect in vivo. For this study, we incorporated the extract in either an oil-in-water (O/W) or a water-in-oil (W/O) cream, both having a comparable lipid content of about 40%. Hydrocortisone acetate (HCA), 1%, was used as positive control.

## 2. Results

### 2.1. Anti-Inflammatory Effects of a HL Extract In Vitro

**Figure 1:**
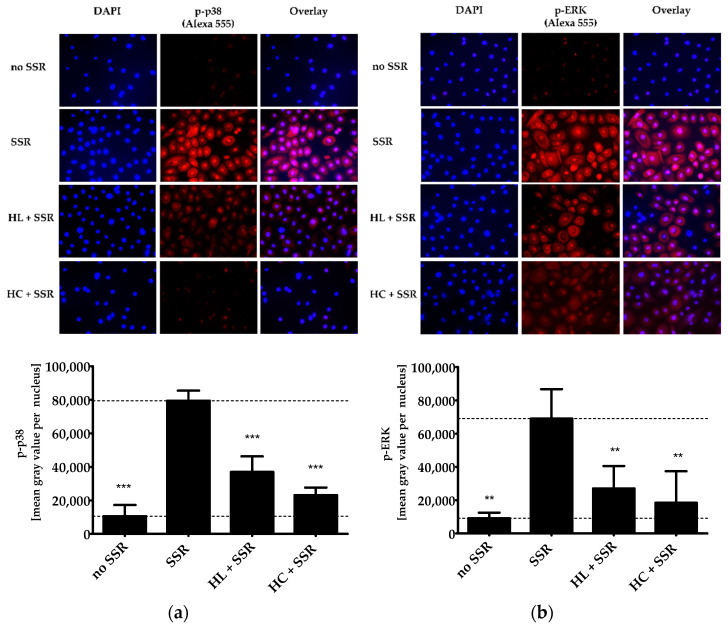
Effect of HL extract on the phosphorylation of p38 and ERK. PHKs were pretreated with HL extract (4 µg/mL) or HC (20 µg/mL) for 2 h and irradiated with 6 J/cm^2^ using a solar simulator. After 20 min, immunofluorescence staining of p-p38 or p-ERK was performed. The nucleus was stained with DAPI. Representative pictures of p-p38 (**a**) and p-ERK staining (**b**) for non-irradiated PHKs (no solar simulator radiation (SSR)), after irradiation (SSR), with HL extract treatment before SSR (HL + SSR), and HC pretreatment before SSR (HC + SSR). The intensity of the staining in the nucleus was measured using the Intensity Ratio Nuclei Cytoplasm Tool (RRID:SCR_018573) in ImageJ. Results of three independent experiments are shown as mean ± SD. The statistical significances were calculated in relation to the SSR-treated sample. ** *p* ≤ 0.01, *** *p* ≤ 0.001.

UVB irradiation leads to the activation and subsequent phosphorylation of the MAPKs p38 and ERK. The so-induced local pro-inflammatory response eventually results in erythema formation [21,22]. To test the anti-inflammatory effect of HL extract, we treated solar simulator-irradiated PHKs with HL extract (4 µg/mL) or the positive control hydrocortisone (HC, 20 µg/mL). This concentration has already been determined in former experiments [1]. The treatment strongly inhibited radiation-induced phosphorylation and nuclear translocation of phospho (p)-p38 and p-ERK (Figure 1a,b). The anti-inflammatory effects of HL extract and HC were comparable with respect to a reduction of the ERK phosphorylation, whereas for the p38 phosphorylation, HC showed a statistically significantly stronger effect than HL extract (*p* ≤ 0.05).

**Figure 2:**
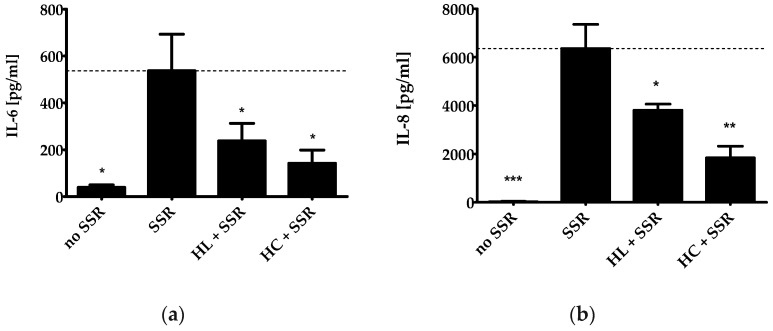
Effect of HL extract on the secretion of IL-6 and IL-8. PHKs were pretreated with HL extract (4 µg/mL) or HC (20 µg/mL) for 2 h and irradiated with 6 J/cm^2^ using a solar simulator. The cell culture supernatant was collected after 24 h of non-irradiated PHKs (no solar simulator radiation (SSR)), after irradiation (SSR), with HL extract treatment before SSR (HL + SSR), and HC pretreatment before SSR (HC + SSR). Then, secretion of IL-6 (**a**) and IL-8 (**b**) was measured using ELISA. Results of three independent experiments are shown as mean ± SD. The statistical significances were calculated in relation to the SSR-treated sample; * *p* ≤ 0.05, ** *p* ≤ 0.01, *** *p* ≤ 0.001.

The activity of these MAPKs has been linked to the expression of the inflammatory cytokines IL-6 and IL-8 [23]. The cytokine IL-6 plays a central role in host defense and elevated levels are a marker for inflammation. The pro-inflammatory chemokine IL-8 is produced by various cell types including keratinocytes to recruit leukocytes (e.g., neutrophils) to the site of radiation-induced skin inflammation [24]. HL extract also reduced the secretion of IL-6 and IL-8 after irradiation of PHKs (Figure 2a,b). The reduction in IL-6 release was not statistically significant between HL extract and HC, whereas with regard to IL-8 release, a significant difference was found, indicating a stronger effect of HC (*p* ≤ 0.05).

### 2.2. Development of Skin-Tolerant and Lipid-Rich Oil-in-Water (O/W) and Water-in-Oil (W/O) Creams with 1% HL Extract

To investigate the anti-inflammatory effect of HL extract topically applied to human skin in vivo, suitable vehicles had to be developed. We chose lipid-rich vehicles to ensure good drug release and penetration as suggested in the literature [6]. Both, an O/W and a W/O vehicle with comparable ingredients and lipid content were developed. The detailed composition is outlined in the Materials and Methods section. Furthermore, we decided to add a high-pressure ethanolic spissum extract from *Gentiana lutea* L. (Gentianaceae) roots (GL extract) to the creams. GL extract has been shown to stimulate lipid and ceramide synthesis in the skin [3]. The lipophilic HL extract with humulone and lupulone as main components had to be integrated into the vehicles together with the hydrophilic GL extract. Furthermore, the two vehicles should have a good skin compatibility and an excellent life cycle assessment (LCA) without microplastics, so that paraffin and PEG as emulsifying system were excluded.

**Figure 3:**
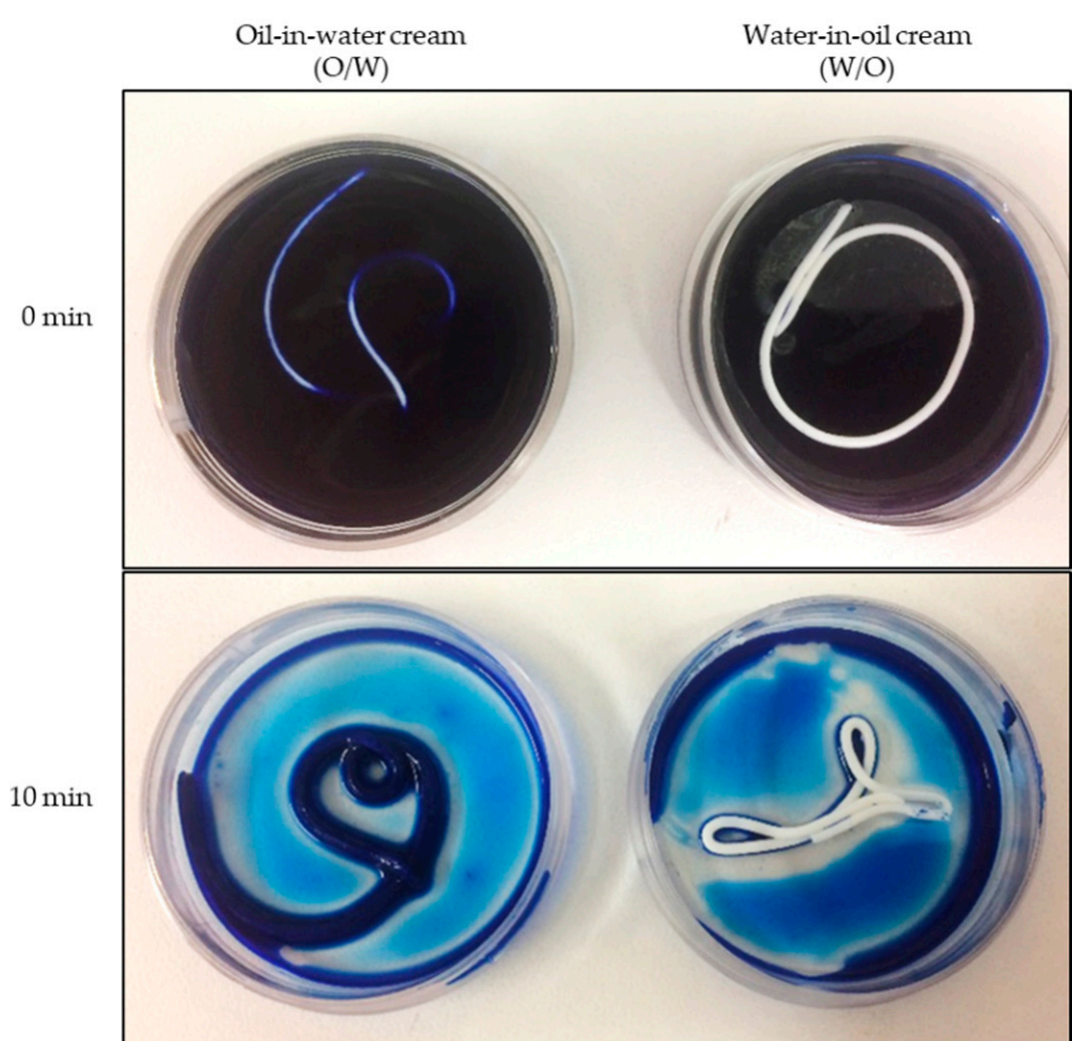
Verification of the O/W and W/O formulation type using methylene blue staining. A small part of both creams was squeezed from the tube into an aqueous 0.1% methylene blue solution. After 10 min, the methylene blue solution was removed and the blue staining of the cream was verified. Blue staining of the creme indicates a O/W formulation.

To verify the emulsion type of the two vehicles, we used the methylene blue staining method. Methylene blue is a hydrophilic substance that stains O/W creams homoge-neously blue but not W/O systems. In Figure 3, it is clearly depicted that the intended emulsion types were confirmed by the staining test.

The galenic stability testing for both creams with 1% HL extract (*w*/*w*) revealed their physicochemical stability as described in the Materials and Methods section.

### 2.3. UVB Erythema Test with 1% HL Extract In Vivo

**Figure 4:**
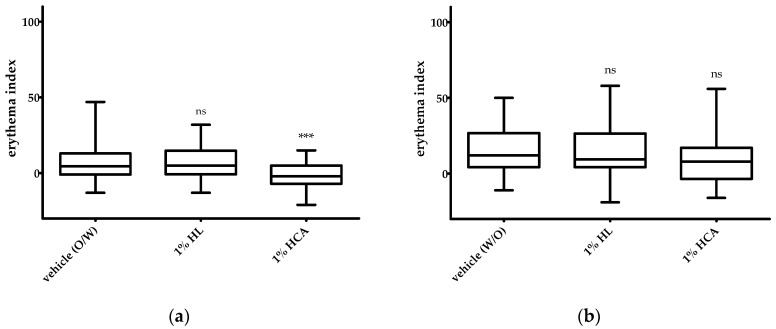
Effect of an O/W cream (**a**) and a W/O cream (**b**) with 1% HL (*Humulus lupulus*) extract or 1% HCA (hydrocortisone acetate) on skin erythema of non-irradiated skin (*n* = 40); The statistical significances were calculated in relation to the vehicle. ns: not significant, *** *p* ≤ 0.001.

The in vivo skin tolerability and the anti-inflammatory effect of 1% HL extract was investigated in the UVB erythema test in comparison to placebo (vehicle) and the positive control, 1% hydrocortisone acetate (HCA). HCA is five times more active in terms of the release and permeation across an animal membrane than hydrocortisone (HC) [25]. The patch test on non-irradiated skin revealed that all investigated creams containing HL extract or HCA were very well tolerated by the skin. No increase in the erythema index was observed compared to the respective placebo vehicle (Figure 4a,b). Vasoconstriction of the skin causing a blanching effect mediated by HCA appeared with the O/W cream but not with the W/O cream (Figure 4a). This effect could be seen in a statistically significant reduction of the skin redness (erythema index). The blanching effect is used to classify the anti-inflammatory potency of topical corticosteroids. From this, we hypothesized that HCA was not sufficiently released from the W/O vehicle.

**Figure 5:**
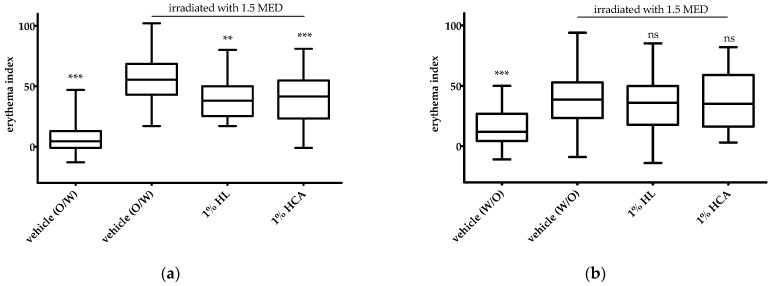
Effect of an O/W cream (**a**) and a W/O cream (**b**) containing 1% HL (*Humulus lupulus*) extract or 1% HCA (hydrocortisone acetate) on skin erythema of human skin irradiated with 1.5- fold MED UVB (*n* = 40); The statistical significances were calculated in relation to the irradiated vehicle; ns: not significant, ** *p* ≤ 0.01, *** *p* ≤ 0.001.

In the UVB erythema test, the actives (1% HL extract or 1% HCA) incorporated in the O/W formulation showed a statistically significant reduction of the erythema (Figure 5a). The anti-inflammatory effects of 1% HL extract and 1% HCA were comparable (Figure 5a). In contrast, neither 1% HL extract nor 1% HCA in the W/O formulation showed an anti-inflammatory effect, i.e., a reduction of the UVB-induced erythema (Figure 5b).

### 2.4. Release and Permeation of HCA from an O/W and a W/O Cream Using Franz Diffusion Cells

To clarify the role of the vehicle, we compared the release rates of an active ingredient from the W/O and the O/W formulation using Franz diffusion cells. HCA was selected as exemplary active ingredient, since it has already been intensively characterized in the literature and was used as positive control in our UVB erythema test [26].

**Figure 6:**
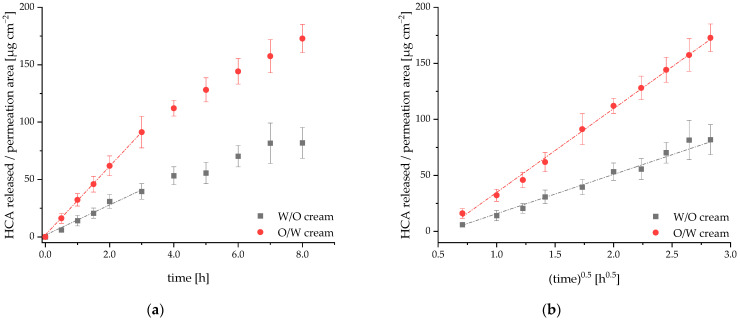
Release profile of HCA from the O/W cream (red dots) and W/O cream (black squares). Profiles were obtained using Franz diffusion cells with polyether sulfone membranes (not rate-limiting) to separate donor cream from acceptor medium (30% ethanol). (**a**) The initial flux of HCA (J_ini_) was determined from the first 3 h of HCA release. (**b**) The amount of released HCA is proportional to the square root of time, i.e., it follows the Higuchi model for drug release from non-erodible matrix systems. Bars indicate standard errors from *n* = 3 independent experiments.

In Figure 6, the release of HCA from the two formulations is compared. For both formulations, the release is linear with time for the first three hours, only. HCA is mainly transported by passive diffusion with Fick’s first law applying under steady state conditions. As polyether sulfone is a high flux membrane for HCA, the steady state was achieved very fast, and no lag phase was observed. We calculated the initial flux J_ini_ of HCA from the first six data points (slope from linear regression). The initial flux was more than two-fold higher from the O/W system (J_ini_ = 30 ± 0.24 µg/cm^2^/h) than from the W/O system (J_ini_ = 13 ± 1.1 µg/cm^2^/h). For later time points, the flux of HCA from the donor vehicle decreased (Figure 6a). Release experiments were performed under sink conditions and infinite dose conditions (not more than approx. 7% of the applied amount of HCA was released from the donor throughout the whole experiment). Therefore, the decrease in flux neither resulted from a saturation of the acceptor medium nor from a complete depletion of the donor. It is best explained by an increasing diffusion distance over time according to the Higuchi model [27]. This model is derived from Fick’s first law for the case of active substances suspended or dissolved in a non-erodible matrix system where a zone of partial depletion migrates into the matrix over time. According to the equations derived by Higuchi, the amount of active substance released per permeation area is proportional to the square root of time. This was confirmed for the release from both formulations (Figure 6b) and is in accordance with results published by Fini et al. [25]. They investigated the release of HCA from four different vehicles. For all vehicles, the amount of HCA released was a function of t^n^ with *n* ranging from 0.50 to 0.56.

Consequently, after a sampling time of 8 h, 173 ± 12 µg/cm^2^ and 82 ± 13 µg/cm^2^ of HCA were released from the O/W cream and the W/O cream, respectively (Figure 6). From this, we conclude with statistical significance (*p* = 0.04) that the release of HCA from the O/W vehicle is much faster than from the W/O system.

## 3. Discussion

Severe inflammatory dermatological disorders, such as psoriasis and atopic dermatitis, can be treated with monoclonal antibodies directed against specific inflammatory cytokines [28,29]. Mild and moderate inflammatory skin diseases, however, are predominantly treated with anti-inflammatory topical products. Their efficacy primarily depends on the pharmacological profile of the active ingredient. Furthermore, it is well established that the vehicle also strongly influences the efficacy and tolerability of topical formulations [7]. Prescription topical medications for inflammatory eczema have primarily included corticosteroids. They quickly reduce symptoms, such as pruritus and erythema. With long-term use, however, they may cause side effects, such as skin fragility or skin irritation [30]. Herbal extracts often possess several active compounds so that they act on various targets simultaneously [31]. St. John’s wort (*Hypericum perforatum* L.), licorice (*Glycyrrhiza glabra* L.), and tormentil (*Potentilla erecta* (L.) Raeusch.) showed promising results in the treatment of atopic dermatitis patients. In the case of psoriasis, barberry bark (*Mahonia aquifolium* (pursh) Nutt.), indigo (*Baphicacanthus cusia*, Brem.), turmeric (*Curcuma longa* L.), olibanum (*Boswellia serrata*, Triana and Planch.) and St. John’s wort (*Hypericum perforatum* L.) were successfully tested in controlled clinical studies or in scientifically sound preclinical studies [31]. Here, we could show anti-inflammatory effects of a humulone- and lupulone-enriched HL extract on irradiated PHKs. As marker for inflammation, we selected the cytokine IL-6 that plays a central role in host defense, as well as the pro-inflammatory chemokine IL-8. It has already been demonstrated that 1 to 5 µM of humulones and lupulones effectively inhibit the tumor necrosis factor alpha (TNF-α)-induced production of IL-6 in the mouse fibroblast cell line L929 [32]. When inhibiting the radiation-induced production of IL-6 with our HL extract, we applied similar concentrations of humulone and lupulone, since 4 µg/mL of HL extract roughly corresponds to 5 µM of humulones/lupulones. Interestingly, combining hop bitter acids with an agonist for glucocorticoid receptor (GR)-α resulted in an additive inhibition of NF-κB activity after treatment with TNF-α [32]. This may point to new options for anti-inflammatory therapy with fewer side effects as the concentration of the GR-α agonist could be reduced. Furthermore, topical administration of hop acids in mice inhibited acute local inflammation in vivo [32]. In addition, the anti-inflammatory effect of the HL extract is in general regarded as safe in terms of side effects due to its long-standing use in brewing and as herbal medicine.

In the present study, we evaluated the anti-inflammatory potential of this humulone- and lupulone-enriched HL extract in human volunteers using the UVB erythema test. This is an established and validated test model to investigate the anti-inflammatory effect of topically applied test substances on human healthy skin in vivo [33]. From our experience, 500 to 1000 times higher concentrations have to be used in vivo compared to in vitro experiments, because the skin acts as a penetration barrier [34]. That is why we incorporated HL extract at a concentration of 1% in the vehicles (10 mg/mL), which is 2500 times higher than the effective concentration in vitro (4 µg/mL).

In chemically induced skin tumor models in mice, topically applied humulone could effectively reduce local inflammation in rodent skin [18,35]. However, in these studies humulone was dissolved in either DMSO or acetone and then topically applied to the dorsal shaved area of the mouse skin. Thus, the active ingredients had not to be released from a vehicle, but instead were dissolved in a penetration-enhancing or even skin-irritating solution. In our human UVB erythema test, the HL extract was incorporated in two different galenic formulations, an oil-in-water (O/W) and a water-in-oil (W/O) cream. *Gentiana lutea* (GL) extract was added to both vehicles although it does not show any anti-inflammatory activity by itself (data not shown). However, it has been demonstrated that GL extract increases lipid synthesis in human keratinocytes in vivo [3,36], and modulates ceramide synthesis in keratinocytes [3]. In the therapy of inflammatory skin diseases, GL extract should improve certain associated skin disorders, such as disrupted epithelial barriers or altered lipid compositions. Both types of vehicle should meet the high standards for natural cosmetics, be well tolerated and thus suitable for long-term use. Particularly in the case of W/O formulations, the incorporation of ingredients with higher polarity can lead to destabilization. Thus, the polarity of the hydrophilic GL extract and the use of plant oils that are in general more polar than mineral oils (hydrocarbons) posed a major challenge in developing the W/O base. A W/O system, however, provides better skin protection or skin barrier protection since the outer phase of the W/O cream is lipidic. This is especially beneficial for the treatment of eczema with disease-related damages to the skin barrier. On the other hand, W/O formulations often leave an oily film on the skin that negatively affects patient compliance. Therefore, a second vehicle was developed as an O/W formulation with comparable ingredients and approximately 40% lipids. After adding HL extract or HCA as active compounds, both the W/O and O/W cream were tested in the UVB erythema test. We compared the two formulations not only regarding their galenic stability but also in terms of their allergenic potential and how pleasant they felt on the skin. Both vehicles were developed on a sustainable ecological basis without microplastics. Hence, common synthetic emulsifiers, binders, and emulsion stabilizers, such as polyethylene, polypropylene, nylon and acrylates, could not be used. Microplastics are solid and insoluble synthetic polymers (plastics) with a size of less than five millimeters. They were avoided because they are made from petroleum in an energy-intensive process that comes along with considerable emission of CO_2_, a greenhouse gas with a severe environmental impact. In addition, microplastics bind pollutants in water which are then taken up by organisms and exert toxic effects [37]. For example, such bound pollutants cause intestinal inflammation, oxidative stress and disruption of the metabolome and microbiome in zebrafish [37,38,39].

Surprisingly, only the O/W cream showed an anti-inflammatory effect in vivo in the UVB erythema test. To clarify the reason for this observation, we first compared the release rates of 1% HCA from the O/W cream and the W/O cream using Franz diffusion cells [40]. It was not possible to investigate HL extract in the release study, because this extract contains not a single but multiple lead substances, including humulone, adhumulone, and cohumulone as well as lupulone, adlupulone, and colupulone. We chose HCA for the release study since it could easily be detected in the acceptor medium via HPLC, showed sufficient solubility in the acceptor medium and was also used as positive control in the UVB erythema test. In addition, the physicochemical characteristics of HCA relevant to skin penetration, such as molecular mass and lipophilicity, are comparable to humulone, one of the main components of HL extract. In fact, the release of HCA was significantly faster from the O/W vehicle than from the W/O vehicle. After release, the actives need to penetrate the skin to exert a clinical effect. Here, the stratum corneum (SC) represents the main barrier for topically applied drugs [41]. In a preliminary Franz cell study, we compared the penetration of HCA from the two vehicles through Strat-M^TM^ membranes that mimic human skin. Strat-M^TM^ membranes consist of two stratified layers: a non-woven fabric support with large pores, and a polyether sulfone inner layer with smaller pores and additional synthetic lipids. After 8 h, much more HCA had penetrated through the membrane from the O/W cream (47 ± 22 µg/mL) than from the W/O cream (10 ± 2 µg/mL). Although still preliminary, these data indicate that a faster release of HCA is followed by a faster skin penetration in case of the lipid-rich O/W cream. In the following, we will briefly discuss some physical parameters of our active components that are key to skin penetration, namely molecular mass, lipophilicity and solubility in the vehicle. As the corneocytes of the SC are closely packed with keratins, the pore size in the SC is as small as 20 nm. Molecules with a molecular mass of larger than 500 Da cannot passively penetrate into the skin [42,43,44,45,46]. In terms of their molecular masses, HCA (404 Da), humulone (362 Da) and lupulone (414 Da) should penetrate well into the skin. A second important parameter is lipophilicity. Substances with logP (P = partition coefficient octanol/water) values in the range of 1 to 4 show good skin permeability [47]. For the model substance HCA, a logP value of 2.2 was determined [48] and for humulone, the most abundant chemical entity in the hop extract, a clogP value of 3.7 was calculated using Molinspiration Cheminformatics (https://www.molinspiration.com, accessed 26 January 2022). In terms of logP values, both HCA and humulone have a well-balanced lipophilicity/hydrophilicity, i.e., they distribute into both aqueous and non-aqueous compartments. It can be assumed that they distribute well from hydrophilic to lipophilic compartments within the vehicles and from the vehicle into the stratum corneum.

Next, a higher solubility of HCA in the O/W formulation would explain the observed differences in release and preliminary penetration experiments. Since the O/W vehicle contains solubilizing agents such as lysolecithin and lecithin, differences in solubility are likely. However, since solubility was not determined in this study, further experiments are needed to clarify this point.

Recently, Casiraghi et al. compared Hildebrand solubility parameters to estimate the thermodynamic activity of Cannabidiol in different semi-solid and liquid formulations. They used this approach to successfully explain the impact of the vehicle on skin permeation [49]. Although this is a very promising and useful concept, it appears to be limited to formulations that consist of chemically well-defined components. Both formulations used in the present study contain complex substances of natural origin that are heterogeneous mixtures of different chemical entities for which a meaningful Hildebrand solubility parameter is difficult to calculate.

Lastly, a better skin penetration from the O/W formulation may also result from the presence of penetration enhancers. In fact, only the O/W formulation contained propanediol that is known to exert an entraining but not an emulsifying effect. In the W/O vehicle, propanediol was omitted to obtain a stable emulsion.

The above considerations and the release/penetration experiments help to understand why, in spite of the promising anti-inflammatory in vitro results of the HL extract, a statistically significant anti-inflammatory effect in the UVB erythema test could only be demonstrated with the lipid-rich O/W cream.

## 4. Materials and Methods

### 4.1. Plant Extracts, Reagents and Membranes

**Figure 7:**
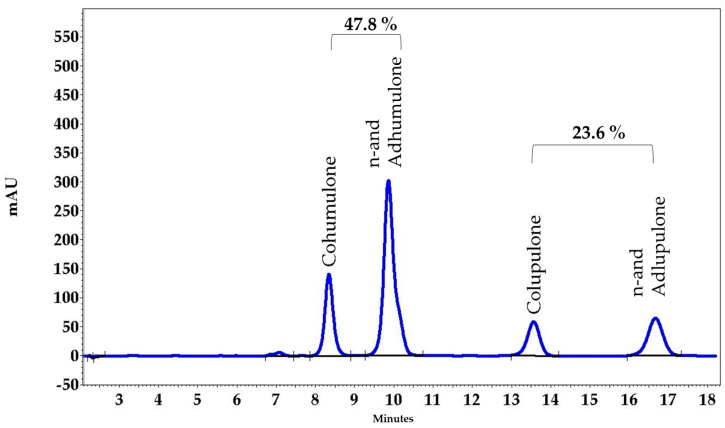
HPLC fingerprint of the HL extract.

The following antibodies and dilutions were used for immunofluorescence staining: rabbit anti-human p-p38 antibody (1:100, Cell Signaling Technologies, Leiden, The Netherlands), rabbit anti-human p-ERK antibody (1:500, Cell Signaling Technologies). The fluorescence secondary antibody Alexa Fluor 555 donkey anti-rabbit IgG was from Thermo Fisher Scientific (1:500, Darmstadt, Germany). DAPI (4′,6-Diamidino-2-phenylindole dihydrochloride), hydrocortisone (HC), hydrocortisone-21-acetate (HCA) and methylene blue were purchased from Sigma-Aldrich GmbH (Taufkirchen, Germany). The polyether sulfone membrane was cut from the vacuum-driven disposable bottle top Stericup^®^ filter with 0.22 µm pore size (Millipore, Temecula, MA, USA). The supercritical genuine hop CO_2_ extract was produced by Flavex (Rehlingen, Germany). In brief, hop flowers (*Humulus lupulus* flos) were dried and extracted with high-pressure supercritical CO_2_. This extraction method has many advantages: (i) complete preservation of the lipophilic spectrum of plant constituents without any dilution; (ii) no solvent residues; (iii) low extraction temperatures avoiding chemical decomposition of the ingredients. No polar compounds such as tannins, flavonoids (or other polyphenols), and xanthohumol are present in the extract. The extract contains 47.8% α-bitter acids (humulones including cohumulone and adhumulone), 23.6% β-bitter acids (lupulones including colupulone and adlupulone), and 7.8% essential oils (Figure 7). The *Gentiana lutea* L. (GL) extract is a High Pressure Ethanol (HPE) extract of yellow gentian roots from Flavex obtained as described elsewhere [36].

### 4.2. Cell Culture

Primary human keratinocytes (PHKs) were prepared from healthy adult skin obtained from reduction surgery (approved by the ethics committee of the University Medical Center Freiburg, Certificate No EK432/18) and isolated according to the method of Rheinwald and Green [50] in Keratinocyte-SFM medium (Thermo Fisher Scientific, Darmstadt, Germany). The cells were cultured at 37 °C in a humidified atmosphere with 5% CO_2_.

### 4.3. Solar Simulator

The solar simulator (Model 81 192; Oriel equipped with q 1000 W xenon arc lamp) delivers UVB, UVA and visible light to simulate the radiation of the sun [51].

### 4.4. Immunocytochemistry (ICC)

3 × 10^4^ PHK were seeded in LabTek chamber slides with 8 chambers (Thermo Fisher Scientific, Darmstadt, Germany), incubated overnight and pre-treated with either 4µg/mL HL extract or 20 µg/mL HC for 2 h. A dose of 6 J/cm^2^ total UV radiation was then applied using a solar simulator. Afterwards, PHKs were incubated for 20 min at 37 °C, fixed using 4% formaldehyde at RT for 10 min and permeabilized with methanol at −20 °C for 10 min. Subsequently, the cells were blocked with 5% BSA for 1 h at RT and incubated overnight at 4 °C with the primary antibodies (anti-p-p38 antibody and anti-p-ERK antibody). DAPI was used for nuclear staining. The percentage of total protein located in the nucleus was measured using the Intensity Ratio Nuclei Cytoplasm Tool (RRID:SCR_018573) in ImageJ. Data are shown as mean ± SD of three independent experiments.

### 4.5. IL-6 and IL-8 ELISA

PHK were pre-treated with either 4 µg/mL HL extract or 20 µg/mL HC for 2 h before irradiation with a dose of 6 J/cm^2^ total UV radiation using a solar simulator and then incubated for 24 h at 37 °C. Concentrations of IL-6 and IL-8 in the cell culture supernatants were measured using high sensitivity ELISA kits (BD, San Jose, USA). The assay was performed according to the manufacturer’s protocol. Data were expressed as mean ± SD of three independent experiments from three independent PHK samples.

### 4.6. Topical Study Products and Stability Tests

The vehicle (placebo) and the verum (vehicle plus actives) were developed, produced and provided by BSI (Beauty Science Intelligence GmbH). The vehicle was either an O/W or a W/O cream with natural lipids and an aqueous phase. The composition of the vehicle according to the International Nomenclature of Cosmetic Ingredients (INCI) for the O/W cream was:

Aqua, dicaprylyl carbonate, helianthus annuus seed oil unsaponifiables, glyceryl stearate, propanediol, C12–16 alcohols, butyrospermum parkii butter, simmondsia chinensis seed oil, squalane, euphorbia cerifera cera, pentylene glycol, alcohol, glycerin, palmitic acid, glycerine soja seed extract, hydrogenated lecithin, sodium stearoyl glutamate, tocopherol, xanthan gum, sodium benzoate, triethyl citrate, gentiana lutea root extract, rosmarinus officinalis leaf extract, benzoic acid, helianthus annuus seed oil, citric acid.

The components of the W/O cream were according to the INCI:

Aqua, tridecane, undecane, helianthus annuus seed oil unsaponifiables, squalane, glycerin, oleic/linoleic/linolenic polyglycerides, polyglyceryl-2-oleate, butyrospermum parkii butter, magnesium sulfate, polyhydroxystearic acid, simmondsia chinensis seed oil, euphorbia cerifera cera, pentylene glycol, sucrose distearate, tocopherol, xanthan gum, sodium benzoate, gentiana lutea root extract, alcohol, rosmarinus officinalis leaf extract, benzoic acid, helianthus annuus seed oil, sodium hydroxide.

The actives of the verum were a supercritical CO_2_ extract from *Humulus lupulus* (HL) or hydrocortisone acetate (HCA) as positive control.

The galenic stability of both creams was tested based on the guidelines of the German Cosmetic Society (DGK). For the galenic stability testing, 15 mL plastic tubes and 80 mL amber glass bottles were used. The following storage conditions were investigated: (a) room temperature (20 to 21 °C); (b) climatic cabinet (−5 to 40 °C in a temperature program that changes the temperature every 12 h, KB 400, #02-35947, Binder GmbH, Tuttlingen, Germany); (c) heating cabinet (40 °C, KB 400, #00-19840, Binder GmbH, Tuttlingen, Germany); freezer (−18 °C, GP1486, Liebherr-International Deutschland GmbH, Biberach an der Riß, Germany); and refrigerator (7 °C; VKS 5000 profi line, Liebherr-International Deutschland GmbH, Biberach an der Riß, Germany) according to the Cosmetics Europe guidelines for evaluation of the efficacy of cosmetic products 2008. The test parameters were:

- pH value (measured with a Schott pH Meter, Mess- & Labortechnik GmbH, Hohenfels-Liggersdorf, Gemany);
- Viscosity of the cream (measured with a Brookfield-viscosimeter, model EVDV-II, serial number RT63203 (AMETEK Brookfield, Hadamar-Steinbach, Germany) at 20 ± 1 °C and at 10 U/min; spindle number 7 for the W/O cream and spindle number 6 for the O/W cream);
- Density of the cream (measured with a DMA-38 bending transducer, Anton Paar Group AG, Graz, Austria, and calculated from the measurement of the natural frequency of a flexural oscillator filled with the medium under investigation);
- Centrifuge test (performed with a Sigma 2-16P centrifuge, Sigma Laborzentrifugen GmbH, Osterode am Harz, Germany, by centrifuging the cream for 30 min at 2000× *g*. A stable formulation shows no phase separation);
- Microscopic image test with photo documentation (performed on a ZEISS Axiolab 5 (upright microscope) with a ZEISS Axiocam 208 color camera, Zeiss, Oberkochen, Germany, at 400- fold magnification to get an overall impression of the structure).
- Results of the stability testing are shown in Table 1.

**Table 1:** Stability testing of the creams after 3 months storage time (results from the different storage conditions are within the reported ranges).

Emulsion Type	pH Value	Viscosity	Density	Centrifuge Test	Microscopic Image
O/W	5.62–5.64	54,500–59,000 cP	0.965	No phase separation	Fine distribution of droplets (Figure 8a)
W/O	n.d. (outer phase is lipophilic)	180,000–210,000 cP	n.d. (narrow capillary could be destroyed by such a high viscosity)	Only at 40 °C, a slight oil film was observed	Very fine distribution of droplets (Figure 8b)

n.d.: not determined.

Furthermore, the samples were organoleptically tested in terms of appearance, smell, and haptics. Measurements were performed at day 0, after 4 weeks and after 3 months.

A stability over 30 months at room temperature is assumed for a cream that remained stable over 3 months under the investigated storage conditions examined in the galenic stability testing. Demixing was not observed after 3 months under any of the storage conditions tested. Both creams remained stable without phase separation. Only the W/O cream was slightly firmer after 3 months of incubation in the climatic or heating cabinet compared to the other storage conditions.

The test preparations for the UVB erythema test (placebo, verum and HCA as positive control) were provided as 10 g in blinded plastic tubes with a conical opening. 

### 4.7. UVB Erythema Test

The anti-inflammatory effect of the cream with 1% HL (verum) or 1% HCA (positive control) was evaluated using the UVB erythema test. The study protocol was approved by the ethics committee of the University Medical Center Freiburg (Certificate No EK113/19) and written informed consent was obtained from all subjects. Inclusion criteria were healthy volunteers of both sexes with an age above 18 years. Exclusion criteria were a severely reduced general condition and participation in another clinical trial within the last 4 weeks. Furthermore, volunteers with dermatological diseases requiring systemic therapy, with known hypersensitivity to one of the components of the topical study pro-ducts as well as necessary concomitant medication with substances that have anti-inflammatory, immunomodulatory or antibiotic effects (e.g., antibiotics, antihistamines, steroids, or immunosuppressants) were excluded. The UVB erythema test was performed as described [33]. In brief, after determination of the minimal erythema dose (MED), the irradiation dose was individually calculated for each volunteer (1.5-fold MED). The skin redness reflects the increased blood flow in dermal vessels and is thus an important parameter for the inflammatory state of the skin. Background erythema (T0) was measured in all test areas before treatment using a Mexameter^®^ MX 16 (Courage & Khazaka Electronics GmbH, Köln, Germany). The measurement is based on absorption and reflection of hemoglobin after excitation with defined wavelengths (568 nm and 660 nm). The test areas were then irradiated with the 1.5-fold MED. Subsequently, the test preparations were applied under occlusive conditions on the test areas using Finn chambers (1.8 cm^2^, Almirall Hermal GmbH) and were fixed with Fixomull^®^ stretch (6 × 4 cm, BSN Medical GmbH, Germany). After 48 h, the test substances were removed and, after a resting phase of 30 min, the skin redness was measured for a second time (T1). Then, the erythema index (T1-T0) was calculated. This allows assessing the anti-inflammatory effect of the preparations compared to the vehicle without actives. At the same time, the test preparations were applied to the non-irradiated side (occlusive patch test) and the fields were also measured using the Mexameter^®^ in order to determine whether the preparations have good skin tolerance in the epicutaneous test without increased erythema index compared to the vehicle. The study was a monocentric, randomized, double-blind study. There were three dropouts who developed no erythema in the initial MED-determination. The study population consisted for the W/O creams of 27 women and 13 men, aged between 20 and 62 years (mean age of women: 33.4 ± 12.6 years, mean age of men: 31.5 ± 9.2 years, overall mean age: 32.8 ± 11.3 years). The mean MED was 80.4 ± 23.9 mJ/cm^2^. The study population with the O/W creams consisted of 25 women and 15 men, aged between 21 and 63 years (mean age of women: 35.5 ± 15.4 years, mean age of men: 27.0 ± 5.1 years, overall mean age: 32.3 ± 13.2). The mean MED was 94.2 ± 24.1 mJ/cm^2^. The background erythema index for the test area treated with the O/W vehicle was low (7.1 ± 11.6) compared to the irradiated O/W vehicle test site (55.5 ± 17.4). Similarly, the background erythema index of the W/O vehicle was low (15.4 ± 14.3). As expected, UVB irradiation increased the erythema index (39.2 ± 24.2).

### 4.8. Drug Release Study

To measure the release of HCA from the vehicles, vertical Franz diffusion cells with a nominal volume of the acceptor compartment of 12.3 mL and a diffusion area of 2.01 cm^2^ were used. They consisted of a small donor compartment separated by a membrane from an acceptor chamber with magnetic stirrer. The acceptor fluid was 30% ethanol in PBS. We used a polyether sulfone membrane (pore size 0.2 µm) as a non-rate-limiting barrier, just to separate the donor from the acceptor compartment [52].

Furthermore, PBS used in the acceptor medium was degassed to avoid formation of bubbles. The concentration of HCA in the acceptor fluid did not exceed 10% of its saturation level (sink conditions) to minimize interference with the free diffusion process [43]. For the donor compartment, an amount of 500 mg of each formulation with 1% HCA (*w*/*w*) was initially applied in Finnchambers that were placed upside down on the membrane. The experiments were conducted in triplicate in a water bath to keep the acceptor medium at 32 ± 1 °C simulating skin temperature. Temperature was measured at the end of the experiment. The polyether sulfone membrane was presoaked with acceptor medium for 30 min to avoid volume loss due to wetting of the membrane during the experiment [53].

The donor compartment and sample ports were sealed with Parafilm^®^ to avoid spilling and evaporation. Aliquots of 200 µL were withdrawn after 0.5, 1, 1.5, 2, 3, 4, 5, 6, 7, and 8 h and subsequently replaced with prewarmed acceptor medium. Samples were analyzed by means of HPLC to quantify the released HCA. Data was plotted and analyzed by linear regression using OriginPro, version 2021 (OriginLab Corporation, Northampton, MA, USA).

### 4.9. HPLC Determination

**Figure 9:**
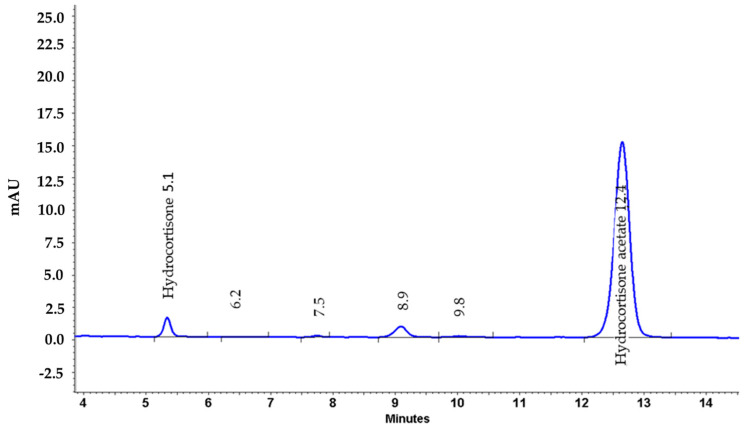
HPLC fingerprint of HCA from the acceptor medium.

Hydrocortisone 21-acetate (HCA) was analyzed from the acceptor medium via a reversed-phase (RP) high-performance liquid chromatography (HPLC) method using HCA as reference substance. The HPLC instrument was a VWR Hitachi Elite LaChrom system consisting of autosampler L-2200, pump L-2130, PDA detector L-2455 and column oven L-2350; data acquisition software EZChrom Elite, version 3.3.2 SP2, build 3.3.2.1037. Separation was achieved using a Lichrospher 100 RP-18e, 250–254 mm column with pre-column 4-4 mm (Merck KGaA, Darmstadt, Germany). Under isocratic conditions, a mixture of 65% water and 35% acetonitrile (Th. Geyer GmbH & Co. KG, Renningen, Germany) was used as eluent (flow rate: 1.0 mL/min; run time: 18 min; injection volume: 20 µL; column temperature: 40 °C; detector wavelength: 238 nm). Figure 9 shows an example chromatogram.

### 4.10. Statistical Analysis

Data analysis was performed using GraphPad Prism version 5.03 software (GraphPad Software, San Diego, CA, USA). Significant statistical differences of the in vitro data were evaluated using one-way analysis of variance (ANOVA) followed by the Newman–Keuls Test. *p* values of *p* < 0.05 were considered statistically significant. Data are presented as arithmetic mean ± standard deviation (SD) of at least three independent experiments from three skin samples. Statistical analysis of the in vivo UVB erythema study was performed with one-way analysis of variance (ANOVA) followed by Bonferroni’s Multiple Comparison Test. For the tests with Franz diffusion cells, the statistical analysis of the results was performed by one-way ANOVA with Bonferroni post hoc test. *p* values are indicated in the figures by asterisks (* *p* ≤ 0.05, ** *p* ≤ 0.01, *** *p* ≤ 0.001).

## 5. Conclusions

We have shown that 1% HL extract displays anti-inflammatory effects comparable to 1% HCA when incorporated in our O/W cream but not our W/O cream. This is probably due to poor release of the actives from the W/O system.

## Figures and Tables

**Figure 8:**
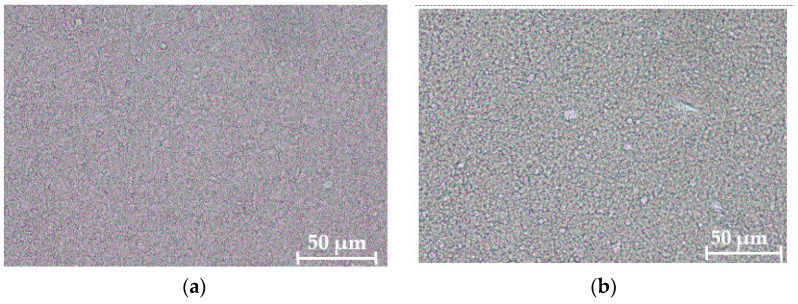
Microscopic images of the O/W (**a**) and W/O (**b**) cream at a magnification of 400×.

## Data Availability

Data is contained within the article.
